# Supplementary material for: Combining ambitious climate policies with efforts to eradicate poverty
Source: Nat Commun. 2021 Apr 27;12:2342. doi: 10.1038/s41467-021-22315-9 (PMC8079366; doi:10.1038/s41467-021-22315-9)
Supplement: Supplementary file 1 — Supplementary Information [file 41467_2021_22315_MOESM1_ESM.pdf]

Combining ambitious climate policies with efforts to eradicate poverty

Bjoern Soergel*[1], Elmar Kriegler[1], Benjamin Leon Bodirsky[1], Nico Bauer[1], Marian Leimbach[1] and Alexander Popp[1]

[1]Potsdam Institute for Climate Impact Research, Member of the Leibniz Association, PO Box 601203, 14412 Potsdam, Germany

Supplementary Information

Contents

1 Detailed description of REMIND-MAgPIE 2
1.1 REMIND . . . 2
1.2 MAgPIE . . . 2
1.3 Coupled model framework . . . 3

2 Details of scenario setup 4

3 Policy cost details 4
3.1 Increased energy and food expenditures . . . 4
3.2 Carbon pricing revenue . . . 5
3.3 Policy costs and revenues . . . 6

4 Derivation of the distributional framework 6

5 Distributional effects of climate policy costs 8
5.1 GDP loss . . . 8
5.2 Increased energy expenditures . . . 9
5.2.1 Income elasticity . . . 9
5.2.2 Empirical estimation . . . 9
5.3 Increased food expenditures . . . 12

6 Monte Carlo simulation for the income distribution in the policy scenario 13

7 Intermediate result: country-level average incomes and Gini coefficients 13

*Corresponding author. Email: soergel@pik-potsdam.de

8 Regression model for poverty outcomes **15**
8.1 Model setup 15
8.2 Data sources 16
8.3 Model parameters 16

9 Uncertainty estimates **17**
9.1 Absolute numbers 17
9.2 Difference between baseline and policy scenario 18

10 Dependence on stringency of the mitigation target **19**

11 Sensitivity analysis: No rescaling of food prices **21**

1 Detailed description of REMIND-MAgPIE

1.1 REMIND

In its default version the Regional Model of Investments and Development (REMIND) [1, 2] describes the global economy with 12 world regions. Large economies (the USA, India, China and Japan) are represented individually, smaller countries are grouped together into aggregate model regions. The regions assemble countries with broadly similar geographical and economical properties, and are harmonized between REMIND and MAgPIE. A list of model regions is given in the caption of Suppl. Fig. 2; for a map showing the region grouping see [3]. For every region REMIND describes the macro-economy by a Ramsey growth model. The main production factors capital, labour and energy are converted into economic output using a production function with constant elasticity of substitution. The demand for energy links the macro-economy to the energy system, which represents a large number of primary energy sources and technological options for conversion into final energy.

In every region, inter-temporal welfare is maximized to determine the optimal investments into the macro-economic capital stock as well as into the energy system. Regions can trade primary energy carriers as well as the composite good; the global (non-cooperative) solution clears all respective markets. REMIND tracks emissions of CO_2 from both fossil fuels and land use, and additionally also other greenhouse gases (GHG) such as CH_4 and N_2O. Emissions are translated into an increase in global mean temperature using the simple climate model MAGICC [4]. The source code and a detailed documentation of REMIND are available online at `https://github.com/remindmodel/remind`; here we use version 2.1.1 [5]. For processing the REMIND (and MAgPIE) outputs, we use a variety of R libraries, which are available open source at `https://github.com/pik-piam`.

1.2 MAgPIE

The Model of Agricultural Production and its Impact on the Environment (MAgPIE) 4 is a modular open source framework for modelling land systems (see [3] and references therein), and can be used for creating global and regional long-term scenarios of future food and agriculture [6]. It combines economic approaches of a partial equilibrium model with (bio-)physical constraints at a clustered 0.5° resolution. Spatially explicit yield patterns and irrigation water availability are derived from the global gridded crop, vegetation and hydrology model LPJmL

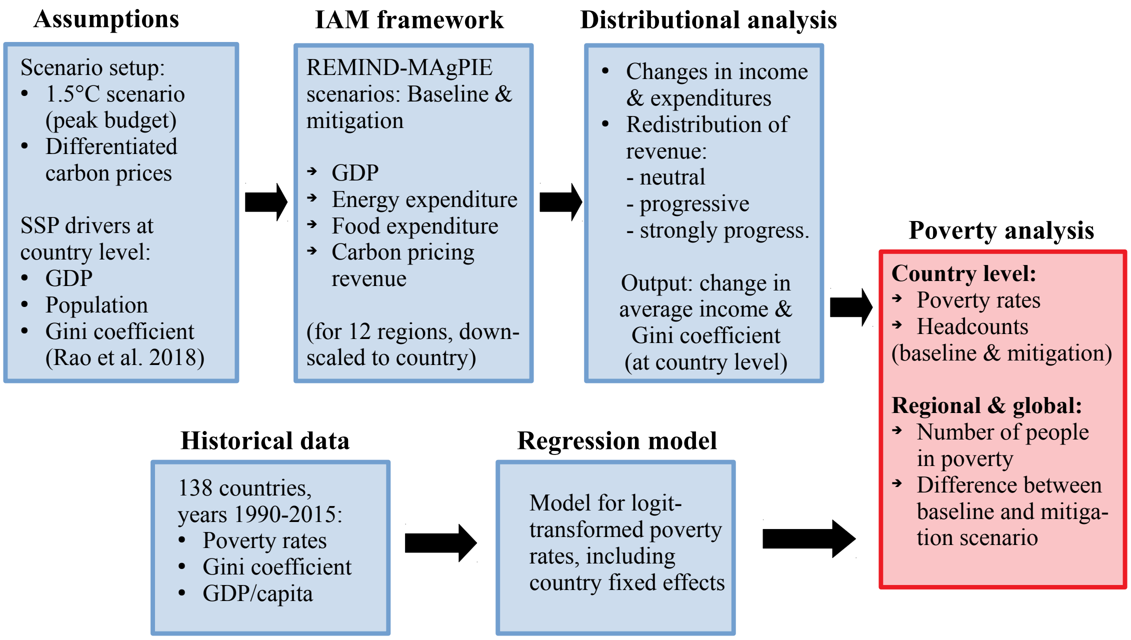

Supplementary Figure 1: Flowchart summarizing the methodology used in this work.

[7]. Agricultural production captures a large number of crop, livestock, processed and informal products; demand distinguishes food, feed, processing, material usage, and food supply chain losses.

The demand for food is determined at the country level accounting for changes in population, demographic structure, body-weight distribution and dietary composition [8]. The latter two are also driven by per-capita income levels, including a reduction of demand through rising prices and therefore decreased real income. Agricultural products can be traded between the 12 world regions that are harmonized with REMIND. Land-use related emissions are derived based on an explicit representation of carbon stocks and the nitrogen cycle, and can be priced with an exogenous carbon price trajectory. The source code and a detailed documentations of MAgPIE are available online at https://github.com/magpiemodel/magpie; for this work model version 4.2.0 [9] is used.

1.3 Coupled model framework

The full integrated assessment modelling (IAM) framework uses an iterative soft-coupling approach to exchange information between the two individual models until a joint equilibrium is reached (see also the short description in the Methods section of the main text). There are several reasons why this coupled system of two large global models is required for our analysis. To begin with, mitigation studies necessarily require a global and economy-wide context. At the same time, a certain degree of regional and sectoral resolution is required to obtain meaningful results. Secondly, also the temporal dimension is important. As mitigation policies will have to tighten over time, the potential for unwanted side effects will evolve. Finally, there is a number of cross-system effects, such as the interlinkage between energy and

land-use systems through bioenergy. Therefore obtaining a holistic and consistent picture necessarily requires that these interlinkages are taken into account.

2 Details of scenario setup

Our mitigation scenarios use a peak-budget approach [10] instead of the end-of-century budgets traditionally used in IAM analyses. The peak-budget approach to some extent avoids scenarios with high levels of mid-century warming and large reliance on carbon dioxide removal in the second half of the century. Our peak budget of 900 Gt CO_2 approximately corresponds to an end-of-century budget of 600 Gt CO_2 (again from 2011 onwards) and is therefore consistent with the assessment in Chapter 2 of the IPCC Special Report on 1.5°C (SR1.5) [11] for a 67% likelihood of stabilizing below 1.5°.

We assume that the carbon price increases linearly until the time of CO_2 neutrality (approximately peak warming). After CO_2 neutrality the price only increases mildly, with a slope sufficient to compensate the small overshoot above 1.5°C. The year of CO_2 neutrality, which separates these two regimes, is determined endogenously with an iterative algorithm.

In addition, we implement a differentiation of carbon prices by development status. Based on their GDP/capita (PPP) in 2015, model regions are assigned a factor between zero and unity that scales their initial carbon price. This factor then converges to unity until 2050; from this point onwards a globally uniform price is assumed.

While the functional form of the regional carbon price trajectories is fixed by these assumption, the price level required to meet the global budget is determined endogenously during the optimization. As an example, the resulting carbon prices for the SSP2 mitigation scenario are shown in Suppl. Fig. 2.

3 Policy cost details

3.1 Increased energy and food expenditures

The additional final energy and food expenditures in model region r and at time step t are computed as

$$\delta_{r,t}^{\mathrm{FE|food}} = \frac{\sum_i \left(\tilde{q}_{r,t}^i \tilde{p}_{r,t}^i - q_{r,t}^i p_{r,t}^i \right)}{\mathrm{GDP}_{r,t}} \tag{1}$$

where q^i and p^i are the quantities and prices (including taxes and subsidies) of the types of final energy/food commodities represented in REMIND/MAgPIE. Outputs from the mitigation policy scenario are denoted with a tilde (e.g. $\tilde{p}$), whereas quantities without a tilde are computed in the baseline scenario.

Note that in particular for food commodities prices can differ substantially between the market for local consumers and the export-oriented market. Typically, food markets in developing countries are divided between a low-priced domestic market and a high-priced export market. Products sold on domestic markets have lower transport and marketing costs, do not need to meet the high products standards of export markets, and in general also have a lower product quality (e.g. broken rice).

As for the purpose of our distributional analysis we need those prices that households are confronted with, we apply a rescaling factor to the prices obtained from MAgPIE (which

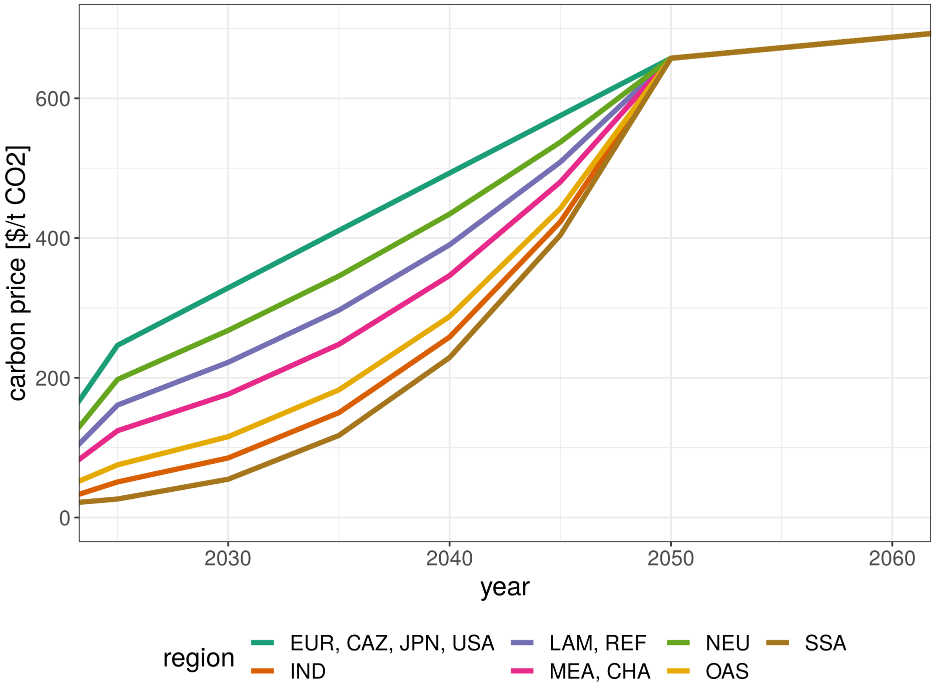

Supplementary Figure 2: Carbon price in our SSP2 1.5°C scenario: the carbon price increases steeply until peak warming is reached (2050 in this case) and then flattens off. The staged accession period lasts until 2050. Model regions that have the same price trajectory according to our differentiation algorithm are shown by only one line. The individual regions are abbreviated as follows: European Union (EUR), Canada, Australia & New Zealand (CAZ), Japan (JPN), United States of America (USA), India (IND), Latin America & the Caribbean (LAM), Russia and former Soviet republics (Reforming Economies, REF), Middle East & North Africa (MEA), China (CHA), non-EU European countries (NEU), Other Asian countries (OAS) and Sub-Saharan Africa (SSA).

resemble the export price). In the absence of country-level data on the price gaps between domestic and international food markets, we assume that this rescaling factor is equivalent to the general MER/PPP ratio. This rescaling factor is particularly relevant for the results in Sub-Saharan Africa, where increased food expenditures form a substantial part of the policy costs in our mitigation scenarios (see Suppl. Fig. 3). We therefore test the sensitivity of our results to this assumption by repeating our analysis without the rescaling. The results of this sensitivity analysis are given in Section 11.

3.2 Carbon pricing revenue

The revenue from carbon pricing that is available for redistribution policies is computed as

$$\delta_{r,t}^{\mathrm{GHG}} = \frac{\sum_j \tilde{e}_{r,t}^{j}\, \mathrm{GWP}^j\, \tilde{\tau}_{r,t}}{\mathrm{GDP}_{r,t}}\,, \tag{2}$$

where e^j and GWP^j are emissions and global warming potentials (100-year time horizon, values from [12]) of the respective greenhouse gas, and τ is the CO_2 price.

Our main analysis considers only the carbon pricing revenues from the energy system as available for progressive redistribution policies. Mitigation potentials in the land-use sector

are more context-specific than in the energy system, and as such implementing and monitoring policies can lead to substantial costs [13]. We thus assume that the revenues are returned to the economy (implemented as a redistribution proportional to income), but unavailable for progressive redistribution. However, we show the effects of including the revenues from pricing CH_4 and N_2O from land use into our progressive redistribution scheme as additional results (see main text). This is especially relevant for the Sub-Saharan African countries where a substantial part of the CO_2eq emissions originates from land use.

The prices for CH_4 and N_2O are capped at the upper end of their respective marginal abatement cost curve [14], leading to maximum prices of around 8,000 \$/t CH_4 and 75,000 \$/t N_2O. As there are no further mitigation options beyond these prices, we assume that a further increase in the CO_2 price does not lead to further increases in the CH_4 and N_2O prices. We do not include any revenues from CO_2 emissions originating from land-use change, as our results from MAgPIE suggest that the net land-use change emissions in our ambitious mitigation scenario are close to zero.

As a proxy for an international climate finance agreement, we also explore a scheme where 5% of the carbon pricing revenues from the OECD countries (approximated by our model regions United States, Japan, Canada/Australia/New Zealand, and the EU and non-EU European countries) are transferred to Sub-Saharan Africa, where they are used for redistribution policies together with the domestic revenue. We focus on this region as a recipient of climate finance, because according to our analysis it is the region with the most substantial negative side effects from ambitious climate policy.

Finally, we note that carbon pricing revenues are only available for redistribution as long as there are positive net emissions. In our analysis, CO_2 neutrality occurs around 2050 in most regions. As we focus on the time horizon until then, the question how negative emission technologies will be financed, and how this affects household incomes, does not play a role in our analysis. The only model region reaching CO_2 neutrality before 2050 in our analysis is Latin America (in 2045). Here we simply set the carbon pricing revenue to zero for the years 2045 to 2050 to avoid a ”negative revenue recycling”.

3.3 Policy costs and revenues

We show in Suppl. Fig. 3 the policy costs and carbon pricing revenues in the SSP2 mitigation scenario for the four model regions where climate policy side effects on poverty eradication are most relevant. The The GDP loss caused by mitigation action and the additional energy and food expenditures determine the reduction in income equivalent for households through climate policy, whereas the revenue from carbon pricing is used for redistribution policies (see above for details).

4 Derivation of the distributional framework

We assume that income in each country is distributed according to a lognormal distribution in the baseline scenario. This functional form has been found to give a good match to measured income distributions for a large sample of countries and years [15, 16]. Furthermore, this choice has the advantages of being completely specified by only two parameters (representing average income and a measure of inequality) and allowing analytical tractability for some of our calculations.

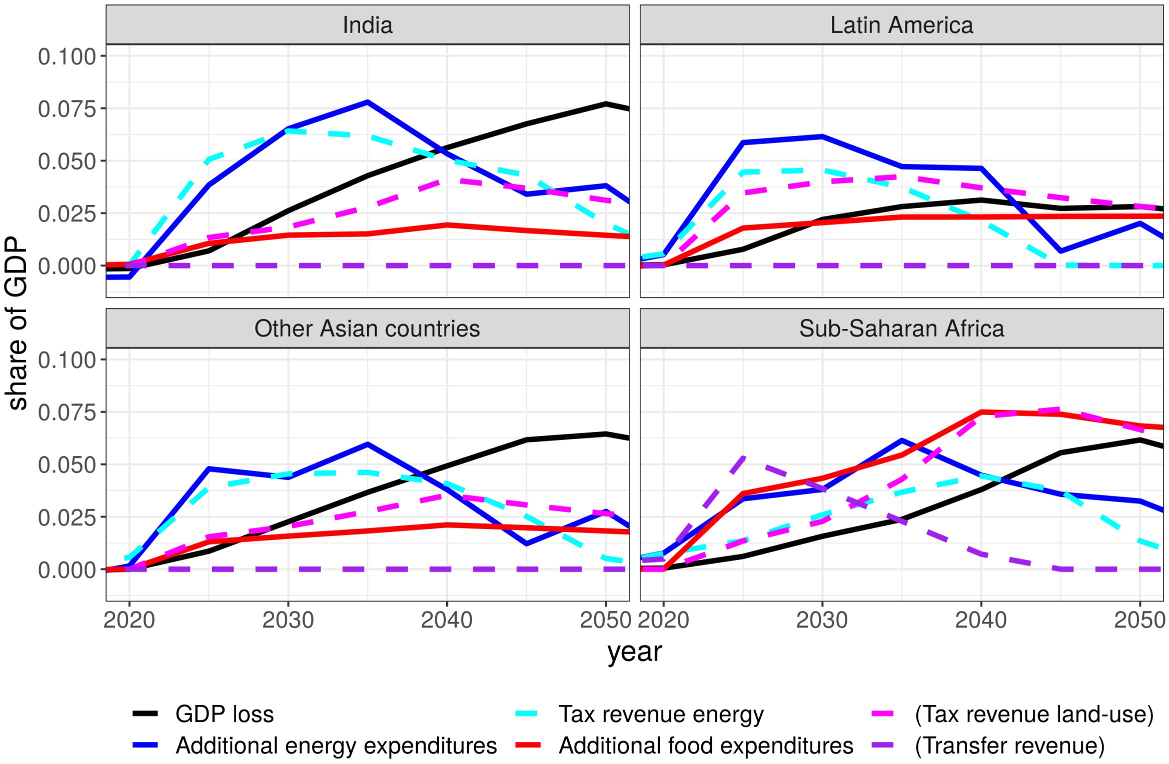

Supplementary Figure 3: Policy costs for the SSP2 mitigation scenario: We show here the different contributions to policy costs (solid: additional energy and food expenditures, GDP loss) and carbon pricing revenue for redistribution (dashed: revenues from energy system, land use, and international climate finance). In our main analysis we only use the revenue from the energy system for progressive redistribution policies. As additional results, we also present the effects of including either the revenues from pricing agricultural emissions, or of a stylized international climate finance scheme. Here we show only the four REMIND-MAgPIE model regions that are most relevant for future poverty outcomes: India (IND), Latin America and the Caribbean (LAM), Other Asia (OAS), and Sub-Saharan Africa (SSA).

The probability density function (PDF) of baseline income y is thus given by

$$p(y) = \frac{1}{y\sigma\sqrt{2\pi}} \exp\left(-\frac{(\log y - \mu)^2}{2\sigma^2}\right) . \tag{3}$$

The parameters μ and σ can be related to the average per-capita income $\bar{y}$ and the Gini coefficient G (ranging from 0 to 1) through [17]

$$\mu = \log \bar{y} - \frac{\sigma^2}{2} \qquad \text{and} \qquad \sigma = \sqrt{2}\Phi^{-1}\left[\frac{G+1}{2}\right] , \tag{4}$$

where Φ is the cumulative distribution function of the standard normal distribution.

Our calculation of the distribution of the income equivalent (or, welfare measured in monetary units) in the climate policy scenario tracks four categories of deviations from the baseline income, capturing both the income and the expenditure side. Here we illustrate the general framework for mitigation costs δ^j (representing one of the δ^j values computed from

the IAM output). We write the income equivalent after the subtraction of mitigation costs as

$$\tilde{y} = [1 - D(y)]y, \tag{5}$$

where $D(y)$ are the *relative* costs for an individual with income y. For $D(y)$ we use the simple power-law form

$$D(y) = A \left(\frac{y}{\bar{y}}\right)^{\alpha_j - 1}, \tag{6}$$

chosen such that the *absolute* costs are $\Delta y = D(y)y \propto y^{\alpha_j}$, and α_j is the income elasticity of mitigation costs for category j.

The dimensionless normalization factor A ensures that costs averaged over all individuals match the national average costs δ^j (again expressed as a share of GDP). The normalization can be computed from the condition

$$\bar{y}(1 - \delta^j) \equiv E[\tilde{y}] = \bar{y} - \frac{A}{\bar{y}^{\alpha_j - 1}} E\left[y^{\alpha_j}\right] \tag{7}$$

using the moments of the lognormal distribution,

$$E[y^{\alpha_j}] = e^{\alpha_j \mu + \alpha_j^2 \sigma^2 / 2}. \tag{8}$$

Combining the above, the costs for an individual with baseline income y are given by

$$\Delta y = \delta^j \, \bar{y} \times y^{\alpha_j} e^{-\alpha_j \mu - \alpha_j^2 \sigma^2 / 2}. \tag{9}$$

The application of Eq. 9 to the different policy cost categories forms the core of our distributional framework.

5 Distributional effects of climate policy costs

For completeness we repeat our equation for the change in income equivalent under climate policy here:

$$\tilde{y} = y - \Delta y^{\mathrm{GDP}} - \Delta y^{\mathrm{FE}} - \Delta y^{\mathrm{food}} + \Delta y^{\mathrm{GHG}}; \tag{10}$$

where y is the baseline income of an individual and the Δ^j terms (positive in our sign convention) are the changes due to climate policy. Importantly, this equation captures changes both on the income and the expenditure side. In the following we detail how we apply the distributional framework developed above to the individual policy cost categories.

5.1 GDP loss

The loss in GDP caused by mitigation action such as carbon pricing reduces the average income of households. We assume that this loss is distributed in a neutral way across income groups, i.e. that it changes only the mean of the income distribution, but not the level of inequality. Therefore we have $\Delta y^{\mathrm{GDP}} = \delta^{\mathrm{GDP}} y$. This is a special case ($\alpha = 1$) of Eq. 9. Additional distributional effects of mitigation policies, such as changes to employment levels or income shares received by (skilled/unskilled) labour and capital, could be incorporated into our framework by specifying the appropriate value of α.

5.2 Increased energy expenditures

For distributing the costs due to rising final energy (FE) prices, we calculate the costs for an individual with baseline income y from Eq. 9.

5.2.1 Income elasticity

For fixing the parameter α_{FE} we first show that under certain assumptions it is identical to the income elasticity of energy expenditures: The income elasticity of additional final energy expenditures for an individual with income y is

$$\alpha_{\mathrm{FE}} \equiv \frac{d\log(\tilde{q}\tilde{p} - qp)}{d\log y} = \frac{d\log(qp)}{d\log y} + \frac{d\log\left[\tilde{q}\tilde{p}/(qp) - 1\right]}{d\log y}\,, \tag{11}$$

where q and p are the final energy quantities and prices, and the tilde denotes the policy run. The second equality separates the income dependence of energy expenditures in the baseline from income-specific demand responses and income-specific price changes. Concerning the income-specific demand response, it can be expected that poorer individuals have a higher price elasticity, and therefore reduce their demand more strongly than higher-income individuals when prices increase. This effect is known to reduce the regressivity (or enhance progressivity) of price increases for example on transport fuels [18].

From a welfare or broader sustainable development perspective, however, it is highly undesirable to have especially the poorest individuals reduce their energy consumption as a response to policy-induced price increases. While this demand response would nominally reduce their income loss, in terms of living standards it would still make them poorer. Here we therefore do not include an income-dependent price response, and further assume that price increases ($\tilde{p}/p$) are independent of income. Under these assumptions α_{FE} reduces to the first term on the right hand side of Eq. 11, which is the income elasticity of final energy expenditures.

5.2.2 Empirical estimation

Given the above we can estimate α_{FE} from household expenditure surveys. For developed countries it is well known that $\alpha_{\mathrm{FE}} < 1$ – in other words, poorer households spend a larger fraction of their budget on energy, and price increases on energy would thus act regressively.

For developing countries the situation is less clear. Using an aggregate data set derived from the World Bank's Global Consumption Database [19], Dorband et al. [20] find that in many developing countries energy expenditures rise slightly faster than proportional with income ($\alpha_{\mathrm{FE}} \gtrsim 1$). Therefore increasing prices even have a slightly progressive effect, although this is largely because the poorest income strata own few energy-consuming devices. Additionally, they partially cover their energy needs with traditional biomass, which is often not sourced from formal markets.

Here we use the same data as in Dorband et al. [20] (kindly provided by the authors of that study). We also employ a similar methodology for estimating the income dependence of the final energy expenditure share, but with a number of relevant modifications. Most importantly, we modify the regression specification as follows:

$$\log\left(\text{FE exp. share}_{c,g}\right) = a + b\log y_{c,g} + c(\log y_{c,g})^2 + \mu_c + \epsilon_{c,g}\,, \tag{12}$$

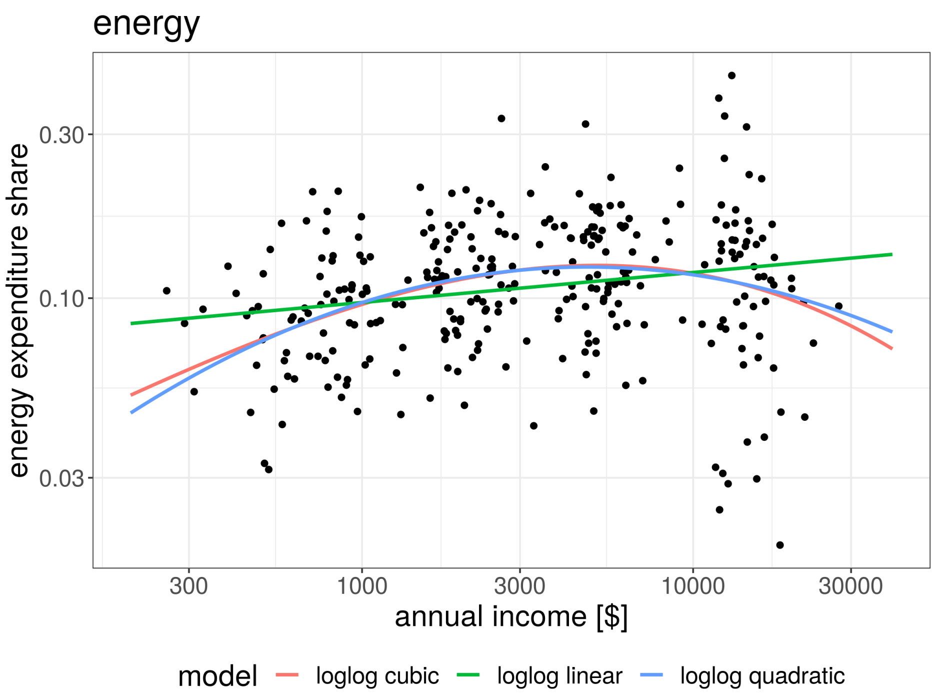

Supplementary Figure 4: Energy expenditure share: We show our estimation of the final energy expenditure share as a function of income, which we use to derive the income elasticity used in the distributional analysis. The inverse U-shape relationship between expenditure share and income indicates that energy price increases are progressive in low-income countries, and regressive in middle/higher-income countries. The scatter in expenditure share at fixed income is partially caused by country-specific conditions (e.g. climate zone, culture), which are absorbed by the country fixed effects in our regression specification. For the model lines shown in this figure, the mean of all country fixed effects was used.

where c denotes a country and g one of the four income groups from the Global Consumption Database (GCD). Using the logarithm of both expenditure share and total expenditures ($y_{c,g}$) increases the sensitivity at low incomes, leads to a slightly increased overall goodness of fit, and simplifies the calculation of the elasticity. The latter relates to the FE expenditure share as

$$\alpha_{\mathrm{FE}} - 1 = \frac{d \log \mathrm{FE\ exp.\ share}}{d \log y} = b + 2c \log y \tag{13}$$

An energy expenditure share that increases with income leads to a progressive incidence ($\alpha_{\mathrm{FE}} > 1$) of price increases, whereas a decreasing energy expenditure share results in a regressive incidence. When the expenditure share as a function of income reaches its maximum at $y_t = \exp(-b/2c)$, the elasticity crosses unity.

Secondly, as we would like to obtain the elasticity as a function of total income (and not total expenditures), we rescale total expenditures from the GCD-based data set with a country-specific consumption share obtained from World Development Indicators [21] (indicator NE.CON.TOTL.ZS). This rescaling has only a very minor effect on the resulting elasticity, as it can largely be absorbed into the country dummies μ_c. Finally, we also convert the expenditure data back to \$ PPP 2005 (as in the original GCD) for later consistency with the SSP GDP scenarios.

Supplementary Table 1: Fitted coefficients of the energy and food expenditure share models, with values in parentheses denoting the respective uncertainties. We also provide the resulting turnover incomes beyond which the expenditure share starts to decrease with income. For income levels above this value energy or food price increases are regressive.

Parameter	Energy: loglog quadratic	Food: loglog quadratic
b	1.64 (0.26)	0.76 (0.17)
c	-0.097 (0.016)	-0.074 (0.011)
turnover income ($ PPP 2005)	4801 $/yr	171 $/yr
adjusted R^2	0.50	0.85

We show our resulting fits for the energy expenditure share in Suppl. Fig. 4, and provide the parameters in Suppl. Table 1. Similarly to Dorband et al. [20] we tested a linear, quadratic (as written in Eq. 12) and cubic model. We also find that the quadratic models performs best according to both Akaike and Bayesian information criteria, and thus use it for the remainder of our analysis. The turnover of the quadratic model is at an average income level of $y_t \simeq 4800$ $ per year, so lower than reported in Dorband et al., largely as a result of the modified regression specification and the conversion to $ PPP 2005. For countries with average income levels below the turnover, energy price increases are progressive, whereas for countries beyond the turnover they are regressive.

As visible in Suppl. Fig. 4 there is substantial scatter in energy expenditure shares at fixed income, partially due to country-specific energy consumption patterns (e.g. climate zone, culture) that are uncorrelated with income. However, this scatter is absorbed into the country fixed effects μ_c, such that our model including the fixed effects captures a reasonable part of the variance in expenditure shares (adjusted $R^2 \approx 0.5$). When calculating the income elasticity according to Eq. 13, the country fixed effects drop out, so that a large effect of this scatter on α_{FE} is avoided. Intuitively this means that the energy expenditure share at fixed income varies significantly between countries, but its fractional change with rising income (as measured by regression parameters b and c) is relatively stable.

Nonetheless we also carefully tested splitting the data into countries with high and low energy taxes and subsidies, respectively. We perform this classification based on the tax and subsidy data compiled by the ADVANCE project [22], and re-estimate our parameters on the individual groups. For example, we find that countries with high FE taxes also have a significantly higher FE expenditure share (i.e., a larger intercept parameter a in our regression), as one would expect intuitively. However, the parameters b and c, which determine the elasticity, do not change at a statistically significant level. In other words, the absolute level of the energy expenditure share depends on the FE tax or subsidy regime, but its fractional change with income is relatively independent of it. As we are only interested in the change with income, we proceed with the estimate from the full data set.

Finally, we also tested including urbanization levels as an additional predictor for energy expenditure shares, but do not obtain a statistically significant dependence on this predictor.

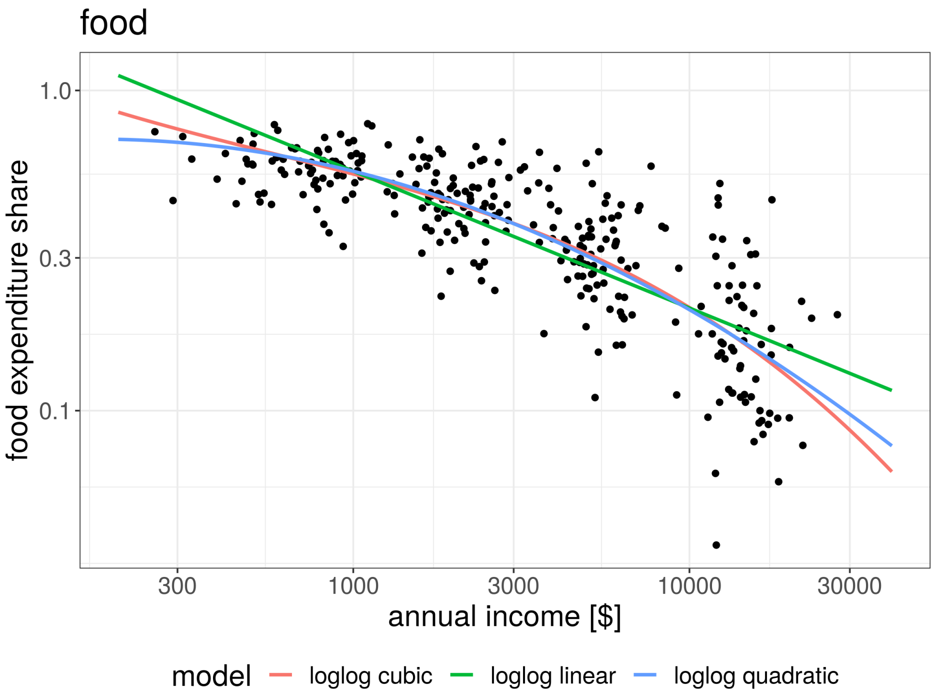

Supplementary Figure 5: Food expenditure share: Same as Suppl. Fig. 4, but for food expenditures. Here we find that food expenditures are regressive at all income levels. The degree of regressivity increases with growing income.

5.3 Increased food expenditures

The costs due to higher food prices are computed in the same way as the additional energy expenditures and depend on the income elasticity of food expenditures, α_{food}. In developing countries, a large fraction of additional income tends to be allocated to food purchases, leading to values around $\alpha_{\text{food}} \simeq 0.8$ [23]. In developed countries, on the other hand, the largest fraction of additional income is spent for other goods, leading to lower values of the income elasticity.

We employ the same estimation technique for the food expenditure share as for final energy, and show the resulting fits in Suppl. Fig. 5. There is a clear trend of decreasing food expenditure share with increasing income, thus food price increases are regressive at all income levels. As found in earlier empirical work [23], this regressivity increases with rising income levels (lower income elasticities). However, food expenditure shares also rapidly decrease with rising income levels, such that the vulnerability against food price increases is particularly high at the lowest income levels.

Again we find that the quadratic model is preferred, its parameters are reported in Suppl. Table 1 alongside the parameters of the final energy expenditure share fit. We further note that the scatter in food expenditure shares is significantly smaller than for final energy, leading to a higher fraction of the variance captured by our regression (adjusted $R^2 \approx 0.85$).

6 Monte Carlo simulation for the income distribution in the policy scenario

For every country and time step, we randomly draw $n = 10^6$ samples from the initial lognormal distribution, which can be thought of as representative individuals.[1] For every sample we compute $\tilde{y}$ from Eq. 10, leading to n samples from the income distribution including the effects of climate policy. This allows us to numerically estimate the new PDF, or to compute any desired summary statistic directly from the samples.

For our application, we are mainly interested in the average income equivalent in the policy scenario, $\bar{\tilde{y}}$, and the new Gini coefficient $\tilde{G}$. Intermediate results for both quantities are shown and discussed in Section 7 below.

The average per-capita income equivalent in the policy scenario is by construction given by

$$\bar{\tilde{y}} = (1 - \delta^{\mathrm{GDP}} - \delta^{\mathrm{FE}} - \delta^{\mathrm{food}} + \delta^{\mathrm{GHG}})\bar{y}\,. \tag{14}$$

Note that in the simplified case of single commodities and fully inelastic demand, both in the energy and land-use sector, we have $\delta^{\mathrm{FE}} + \delta^{\mathrm{food}} = \delta^{\mathrm{GHG}}$, such that the above reduces to $\bar{\tilde{y}} = (1 - \delta^{\mathrm{GDP}})\bar{y}$.

The Gini coefficient in the policy scenario is computed from the ordered samples $\{\tilde{y}_i\}$ as

$$\tilde{G} = \frac{1}{n}\left[\frac{2\sum_{i=1}^{n} i\tilde{y}_i}{\sum_{i=1}^{n}\tilde{y}_i} - n + 1\right]. \tag{15}$$

7 Intermediate result: country-level average incomes and Gini coefficients

As intermediate results of our distributional analysis we show here the effects on average income (Suppl. Fig. 6) and Gini coefficient (Suppl. Fig. 7) in our SSP2 mitigation scenario for four countries which are representative for the respective model regions. Both the change in average income and Gini coefficient affect poverty rates, therefore these intermediate results identify the relevant drivers behind our findings.

The average income equivalent changes under climate policy according to Eq. 14, which captures the effects from both income and expenditure side. We find that losses in the average income equivalent reach up to 5% or less in 2030, and up to 8% in 2050. These substantial losses in average income for developing countries reflect that even our strong differentiation in carbon prices (Suppl. Fig. 2) does not lead to an equal distribution of mitigation costs at the international level [24], highlighting the role of international climate finance in an equitable burden sharing. Indeed the mechanism that we explore as a source of additional revenue for the Sub-Saharan African countries would largely offset their reduction in per-capita incomes.

At the same time, these reductions in per-capita incomes of developing countries also need to be viewed against the effects of unabated warming on economic development. The latter would likely be substantially larger, especially in the second half of the century (e.g. [25]).

In many countries the Gini coefficient is already projected to change in the baseline scenario taken from Rao et al. [26]. The effects of climate policy that we calculate here are in addition to these baseline trends (see Suppl. Fig. 7 for our results in SSP2). In general, we

[1] We have checked that $n = 10^6$ ensures sufficient convergence of our Monte Carlo results.

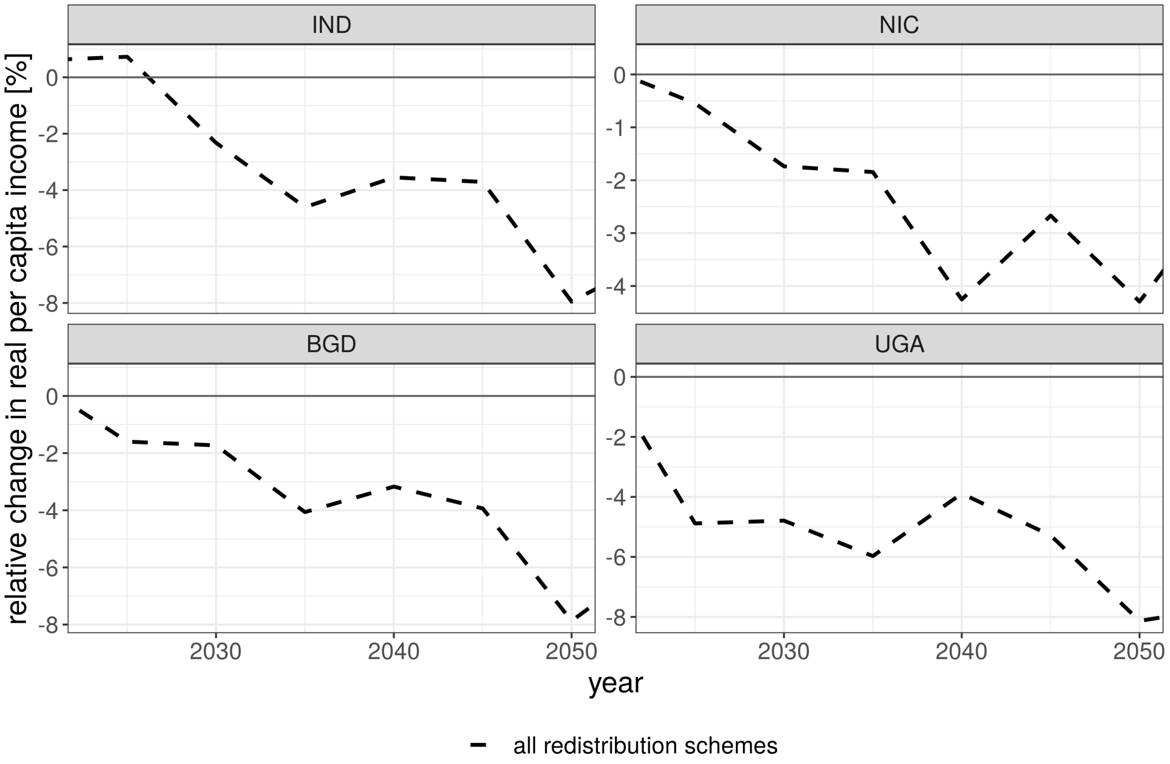

Supplementary Figure 6: Average income equivalent: We show here results for the change in average per-capita income equivalent for a selection of countries from different model regions: India (IND), Nicaragua (NIC), Bangladesh (BGD), Uganda (UGA). The countries are representative for the REMIND-MAgPIE model regions India, Latin America, Other Asia, and Sub-Saharan Africa. All results are expressed relative to our baseline income trajectories taken from the GDP/capita values of SSP2. All redistribution schemes are identical in terms of their effect on the average income, but different in their effect on the level of inequality (see next figure).

find that climate policy without a progressive redistribution of the carbon pricing revenues will increase inequality in all countries. If, however, revenues are used for a progressive redistribution policy, such as the equal-per-capita scheme considered here, this trend is more than compensated in almost all countries, leading to *lower inequality in the case with climate policy*. This reduction of inequality is even stronger when the carbon pricing revenues are redistributed according to our strongly progressive scheme.

Depending on the relative magnitude of additional expenditures and the carbon pricing revenues, the changes to the Gini coefficient caused by climate policy can be significant. For the example of Uganda, we find that climate policies without progressive redistribution would increase the Gini by about 0.02 (i.e., 2 points on a 0-100 scale) by 2050 compared to the baseline, thus reverting the baseline trend of mildly decreasing inequality. A progressive revenue recycling more than compensates this effect, leading to a Gini that is substantially lower than in the baseline case (as long as substantial carbon pricing revenues are available). This behaviour is representative also for many other countries.

The poverty outcomes we show in our main analysis are thus a combination of reduced per-capita income, and increased or decreased levels of inequality (depending on the redistribution

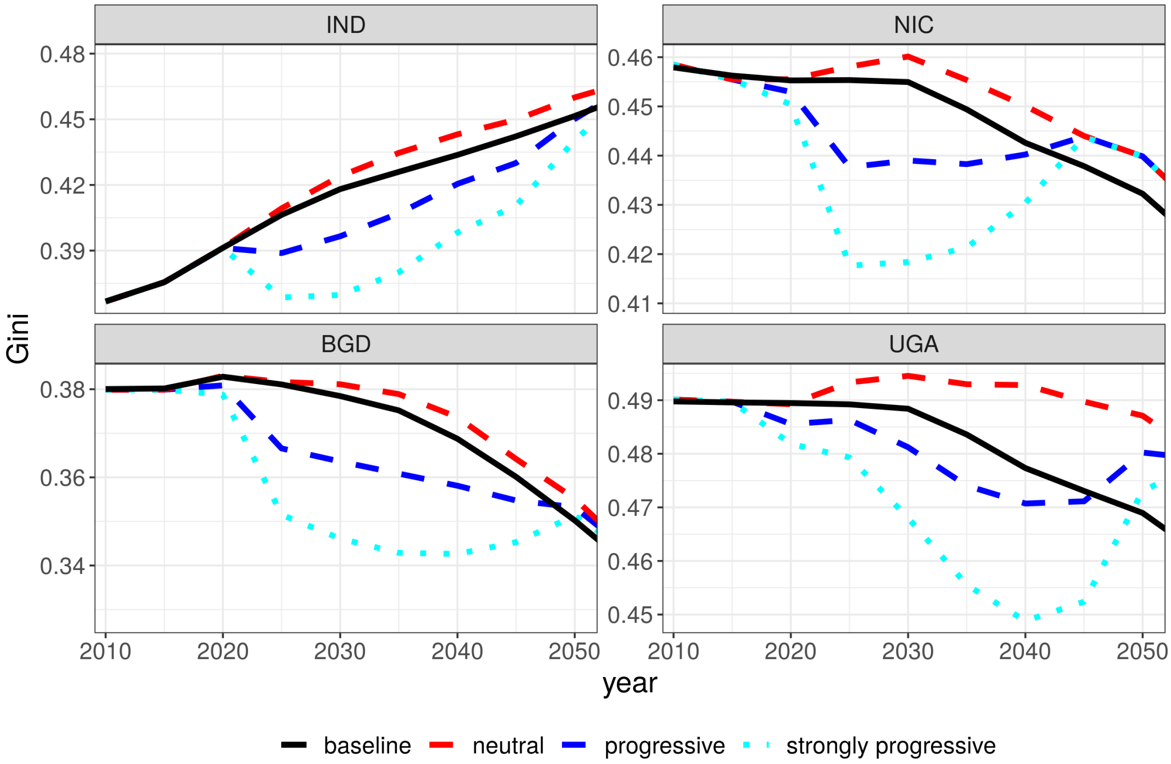

Supplementary Figure 7: We show here results for the Gini coefficient for a selection of countries from different model regions: Bangladesh (BGD), India (IND), Nicaragua (NIC), Uganda (UGA). The baseline projections are taken from the SSP2 Gini projections by Rao et al. [26]; the three policy scenarios with different revenue recycling schemes ('neutral', 'progressive' and 'strongly progressive') are computed according to Eqs. 10 and 15.

scenario). Whether or not poverty side effects of climate policy can be compensated from the revenues depends on the respective magnitude of these different drivers.

8 Regression model for poverty outcomes

8.1 Model setup

We repeat the main regression equation for completeness here. Denoting the share of the population in country c at time t that is *above* the poverty line by $s_{c,t}$, our regression model is specified by

$$\log \frac{s_{c,t}}{1 - s_{c,t}} = \beta_0 + \beta_1 \log \bar{y}_{c,t} + \beta_2 G_{c,t} + \beta_3 \log \bar{y}_{c,t} \times G_{c,t} + \nu_c + \epsilon_{c,t} \, . \tag{16}$$

We first fit the regression model using historical data, and subsequently use the fitted parameters to calculate future projections including the effects of climate policy.

Our dependent variable are the *log-odds* (or *logit*) for an individual to be above the poverty line. This logit transformation maps the share $s_{c,t}$ from a 0–1 range onto an unbounded range,

allowing for a convenient fit with a simple linear model.[2] We include an interaction term between G and $\log \bar{y}$, as well as a country fixed effect (ν_c) to capture idiosyncratic differences between countries (such as national poverty alleviation programs). The residual, country- and year-specific error is denoted by $\epsilon_{c,t}$.

We have also tested other measures of inequality instead of the Gini coefficient in this regression model. In particular we have repeated our fit with the income share of the first decile, which should be particularly relevant for poverty outcomes. We find that the variance in the data explained by this modified model is comparable to our default case with the Gini coefficient. This suggests that our model is robust against the choice of inequality metric used. As future projections are only available for the Gini coefficient [26], we use the latter in our main analysis.

8.2 Data sources

We use historical data from the years 1990-2015 to fit the regression model. The poverty share and the Gini coefficient are obtained from the World Development Indicators [21] (indicators SI.POV.DDAY for the \$1.90 poverty line, SI.POV.UMIC for the \$5.50 poverty line, and SI.POV.GINI, respectively), and are in turn sourced from PovcalNet (March 2020 version, [28]). GDP/capita data in \$ PPP 2005 is taken from [29]. The reason for fitting the regression model with data in 2005 PPP is that the SSP scenarios that we will subsequently use for future projections are also provided in this unit.

The availability of inequality and poverty data varies substantially between countries (see Suppl. Fig. 9 below). While for some countries data is available at least every few years (e.g. China, Indonesia), for other countries only a small number of data points are available over our fit period (e.g. India, Nigeria). In total, our data set comprises 1,160 country-year observations from 131 different countries. Countries with only a single (e.g. Zimbabwe) or no observation (e.g. Afghanistan) over the historical period are removed from our analysis, as no (reliable) determination of the country fixed effect is possible for these countries. Of the countries that are relevant for global poverty numbers (low GDP/capita and/or high poverty rate, significant population) this affects Afghanistan, Haiti, Cambodia, Somalia and Zimbabwe. However, the combined population of these five countries in 2015 was below 90 million, so even if their poverty rates are high, this would not substantially change our global results.

8.3 Model parameters

Our fit indicates that both GDP/cap. and the Gini coefficient are significant drivers for poverty rates in a country. Our simple model explains a large part of the variance in poverty rates (adjusted $R^2 = 0.93$). Even when the country fixed effects are omitted, the model still has substantial explanatory power (adjusted $R^2 = 0.78$). The fitted coefficients are reported in Suppl. Table 2. We show a visualization of the original data and our model in Suppl. Fig. 8. Here we compare the logit values for all countries from the original data to illustrative model predictions as a function of GDP/capita and for different Gini values.

[2] This approach is equivalent to fitting a logistic function directly to $s_{c,t}$. It is somewhat inspired by the findings of Rao and Pachauri [27], who report that progress in metrics of living standards and access indicators approximately follows S-curves.

Supplementary Table 2: Fitted coefficients of the regression model for poverty rates. The p-value stated in the right column is the result of a two-sided t-test for the respective variable.

Variable	Coefficient (standard error)	$p(>\|t\|)$
log(GDP/capita)	3.57 (0.22)	$< 2 \times 10^{-16}$
Gini	15.2 (4.5)	6.5×10^{-4}
log(GDP/capita)×Gini	−2.96 (0.55)	1.0×10^{-7}
Observations	1,160	
R^2	0.938	
Adjusted R^2	0.930	
Residual Std. Error	0.583 (df = 1026)	

Furthermore, Suppl. Fig. 9 shows the original data and the model fit, now converted into poverty rates, for a selection of individual countries. In general, our simple model provides a good fit to the levels and trends of poverty rates in socioeconomically very diverse countries. Also at the global level our model captures the level and trend of recent global poverty data very well (see Fig. 3 in main text).

To provide some illustration for the size of the respective effects, we first consider a doubling of GDP/capita. This changes the log-odds by $\Delta\,\mathrm{logit}\,s = (\beta_1 + \beta_3 G) \times \log 2 = 1.45$ if we take $G = 0.5$ for illustration. Assuming that initially $s = 50\%$ ($s = 90\%$) of the population are above the poverty line, this share changes to 81% (97%) through the doubling of GDP/capita. For comparison, changing the Gini coefficient by $\Delta G = -0.2$ (a substantial reduction in inequality) improves the log-odds by $\Delta\,\mathrm{logit}\,s = (\beta_2 + \beta_3 \log \bar{y}) \times \Delta G = 1.99$ if we take $\bar{y} = 5000\,\$$ for illustration.[3] For the same examples as above, this corresponds to increasing the share of the population above the poverty line to 88% (99%) if initially the share was 50% (90%).

We therefore see that both an increase in national average income as well as a reduction of inequality will substantially decrease poverty rates. If, however, ambitious poverty reduction targets as in SDG 1 are to be fulfilled, both strong and sustained growth in developing countries *and* a substantial reduction of inequality are required (see also [30]).

9 Uncertainty estimates

9.1 Absolute numbers

From the regression model we compute 68% prediction intervals for $\mathrm{logit}(s_{c,t})$ for every country and year. We then use standard uncertainty propagation to infer the corresponding 68% prediction intervals for $s_{c,t}$ and the national, regional and global headcounts. The resulting uncertainties for individual countries are relatively large, typically in the range of $\sigma_{s_{c,t}} \simeq 0.1$. For the global results this translates into an uncertainty of $\sigma_P \simeq 100$ million people in 2015 (compared to a total model prediction of $P \simeq 770$ million).

[3] Note that for all realistic values of GDP/capita, a decrease in G increases the share of the population above the poverty line, as one would expect intuitively.

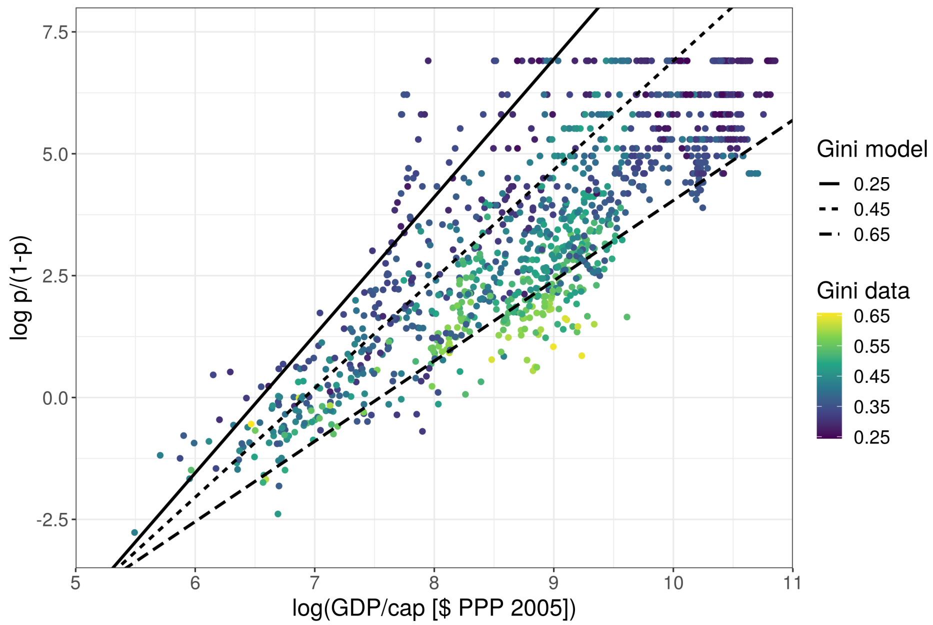

Supplementary Figure 8: In this visualization of our model we show the log-odds (logit) of being above the poverty line as a function of GDP/capita (all countries combined). Log-odds of zero translate to 50% of the population in poverty, moving upwards in this plot corresponds to lower poverty rates. The individual points are colour-coded by the measured Gini values. The data clearly show that at fixed GDP/capita more unequal countries (higher Gini values) tend to have higher poverty rates – as one would expect intuitively. We combine this with illustrative model predictions for three different Gini values; note that for calculating these illustrative predictions we used the country fixed effect of Uganda. The vertical alignment of points in the top right corner is a result of the WDI data being limited to 3 significant digits (e.g.: logit(0.999) = 6.9).

This level of uncertainty does not come as a surprise. All methods to project poverty outcomes rely on estimating certain parameters that describe the distribution of income. An advantage of our approach of directly estimating the relation between average income and Gini and poverty outcomes at the national level is that it allows for a straightforward and transparent propagation of uncertainty.

9.2 Difference between baseline and policy scenario

We also estimate the uncertainties for the difference between policy and baseline scenarios. It is important to note that the uncertainty between the poverty projections in the policy and baseline scenarios are highly correlated, as they are based on the same underlying regression model. We denote the poverty headcounts in policy and baseline scenario by $\tilde{P}$ and P and the corresponding uncertainties by $\sigma_{\tilde{P}}$ and σ_P, respectively. For calculating the uncertainty of the difference $\Delta P = \tilde{P}$ - P, we make the simplifying assumption that the uncertainties between policy and baseline result are fully correlated (i.e. the off-diagonal elements of the correlation matrix are unity). Under this assumption we find

$$\sigma_{\Delta P} = \left|\sigma_{\tilde{P}} - \sigma_P\right| . \tag{17}$$

In other words, due to the correlation between the policy and baseline outcomes the uncertainty of their difference is smaller than their individual uncertainties.

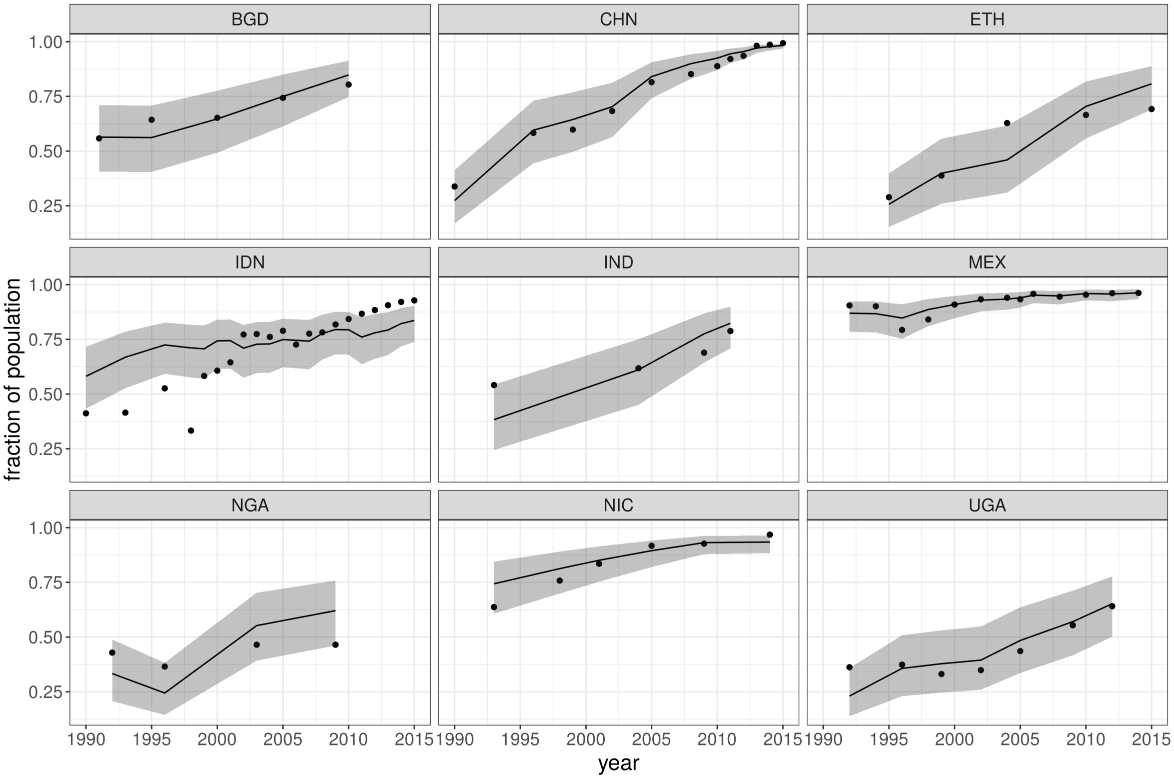

Supplementary Figure 9: We show here the historical data on population shares above the poverty line (points) and the predictions of the regression model (solid lines) for the following countries: Bangladesh (BGD), China (CHN), Ethiopia (ETH), Indonesia (IDN), India (IND), Mexico (MEX), Nigeria (NGA), Nicaragua (NIC) and Uganda (UGA). The shaded bands are 68% *prediction* intervals.

10 Dependence on stringency of the mitigation target

Our main analysis considers an ambitious mitigation scenario consistent with the 1.5°C target. It can be expected that the poverty side effects reduce when relaxing the stringency of the mitigation target. At the same time, however, also the carbon price revenue available for redistribution policies would be reduced. We have therefore repeated our analysis with two less ambitious mitigation scenarios, corresponding to a well-below 2°C and 2°C mitigation pathway. Technically we have implemented the 1.5°C target as a CO_2 budget of 900 Gt for the time from 2011 until the time of CO_2 neutrality. The alternative targets are implemented as budgets of 1100 and 1300 Gt CO_2. We show the results of these additional analyses in Suppl. Fig. 10.

We find that indeed the poverty side effects of mitigation policies reduce significantly for the less stringent target. For our distributionally neutral redistribution scheme we project an increase in the 2030 poverty by just above 30 million people for the well-below 2°C scenario, and by around 25 million people for the 2°C scenario. Again our results show that a progressive redistribution of the carbon pricing revenues is sufficient to (nearly) compensate this increase at the global level, but that there is a strong regional heterogeneity in poverty outcomes. Notably, the most ambitious mitigation scenario performs best in terms of the net change to the poverty headcount after progressive redistribution. On the other hand, in the less

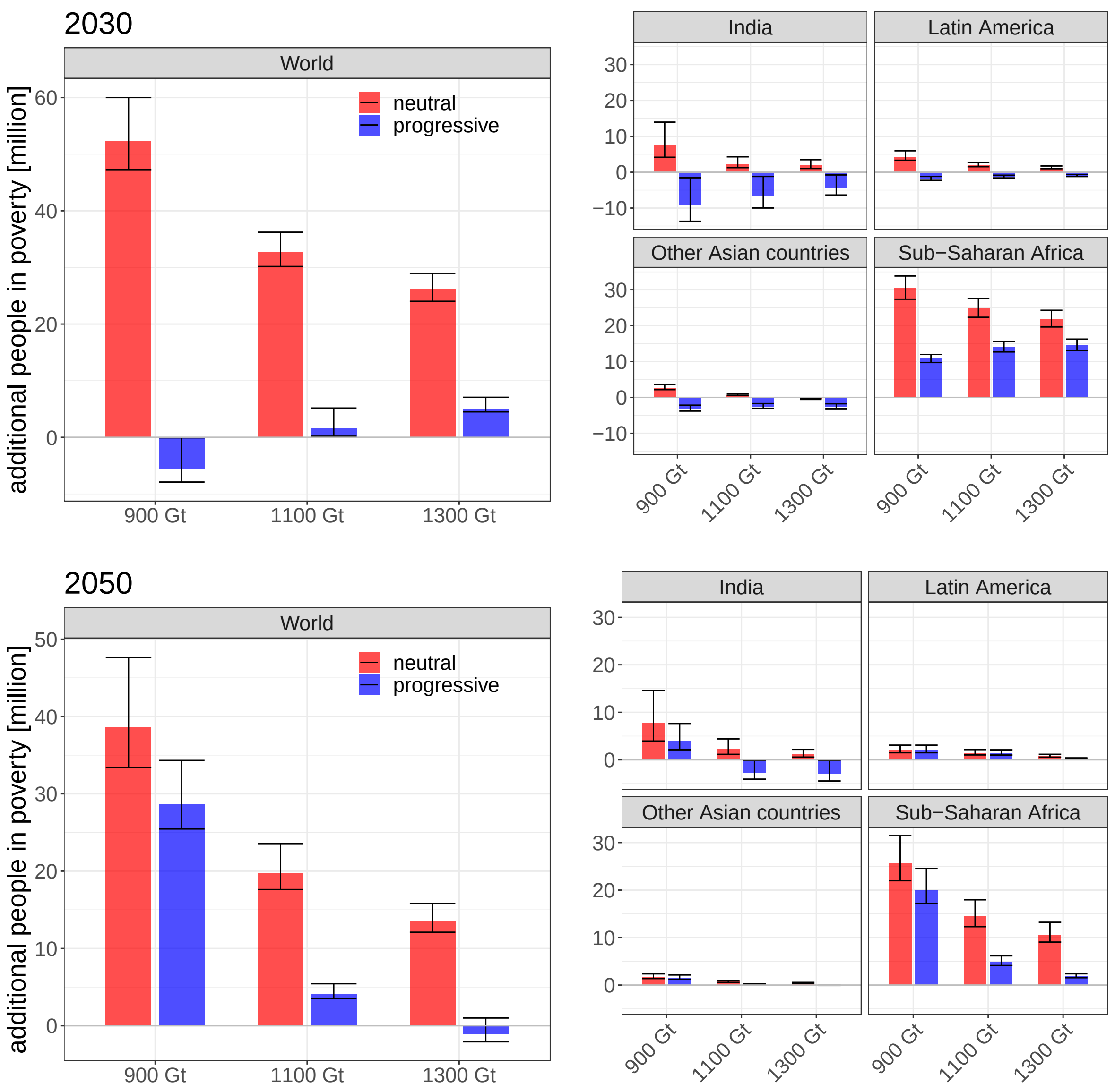

Supplementary Figure 10: ***Additional*** **number of people in poverty:** same as Fig. 4 in the main text, but varying the stringency of climate policy instead of the SSP dimension (here fixed to SSP2). The stated CO_2 budgets correspond to climate targets of 1.5°C, well-below 2°C, and 2°C. The top panels show results for 2030 (as the main figure), bottom panels are for 2050. See the caption of Fig. 4 in the main text for an explanation of the error bars.

ambitious mitigation scenarios it is still possible to largely compensate the policy side effects in 2050, which was not the case for our default 1.5°C scenario. This is a consequence of higher residual emissions and thus carbon pricing revenues in these less ambitious mitigation pathways.

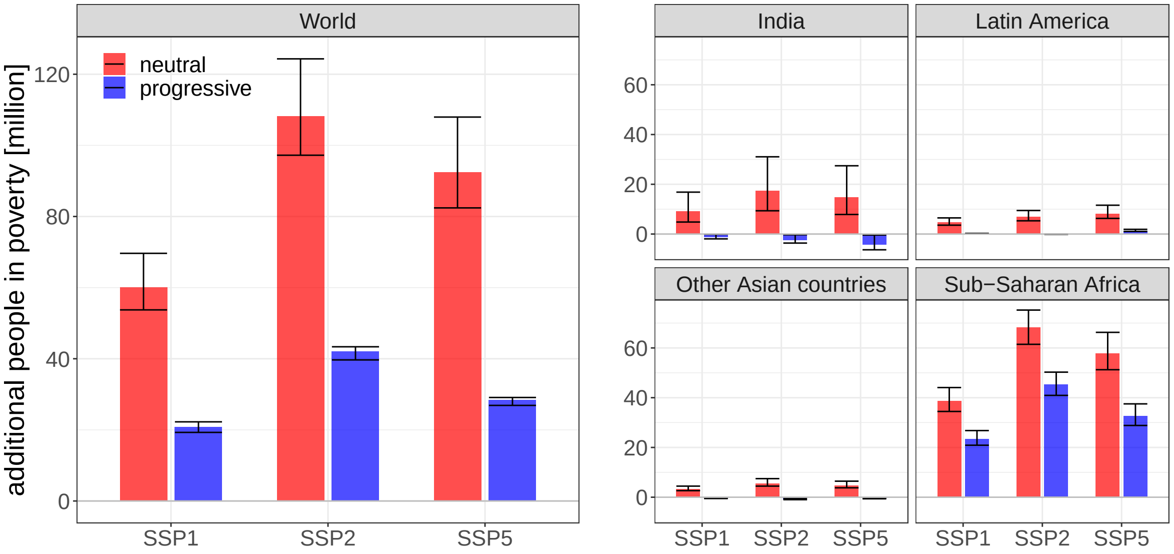

Supplementary Figure 11: *Additional* people in poverty: Same as Figure 4 in the main text, but without the rescaling of food expenditures. See the caption of Fig. 4 in the main text for an explanation of the error bars.

11 Sensitivity analysis: No rescaling of food prices

As discussed in Section 3.1 above, we have rescaled the food prices obtained from MAgPIE with the MER/PPP ratio to obtain prices that better represent the purchasing power of households in developing countries. Here we now test the sensitivity of our results to this rescaling by repeating our analysis without it. In this case the underlying assumption would be that households in developing countries pay world market prices for staple food products, which very likely overestimates the magnitude of distributional effects of changes in food price. For example, this assumption would approximately double the *additional* food expenditures in Sub-Saharan Africa compared to our main analysis, which would then reach an additional 9 percentage points of the average income by 2030 and additional 15 percentage points by 2050. As a result, food prices in SSA would be by far the dominating channel for poverty effects of mitigation policies.

As SSA is also the region with the highest poverty rate, we find a substantially higher poverty side-effect here than in our main analysis where we applied the price rescaling (Suppl. Fig. 11). We project an additional 70 million people in poverty in SSA by 2030 for the neutral redistribution scheme, which is reduced to 45 million in the case of progressive redistribution (both numbers for SSP2). In the other regions food prices are not the dominant channel for distributional effects, and therefore it would still be possible to compensate poverty side-effects from the carbon pricing revenues. As a result, the global figures are largely dominated by the outcomes in SSA.

We have also repeated our analysis with a strongly progressive redistribution and the additional revenues as in the main text, i.e. either from pricing of land-use related CH_4 and N_2O emissions, or from an international transfer scheme (Suppl. Fig. 12). Again focusing on SSA, we find that a strongly progressive redistribution, or alternatively redistributing

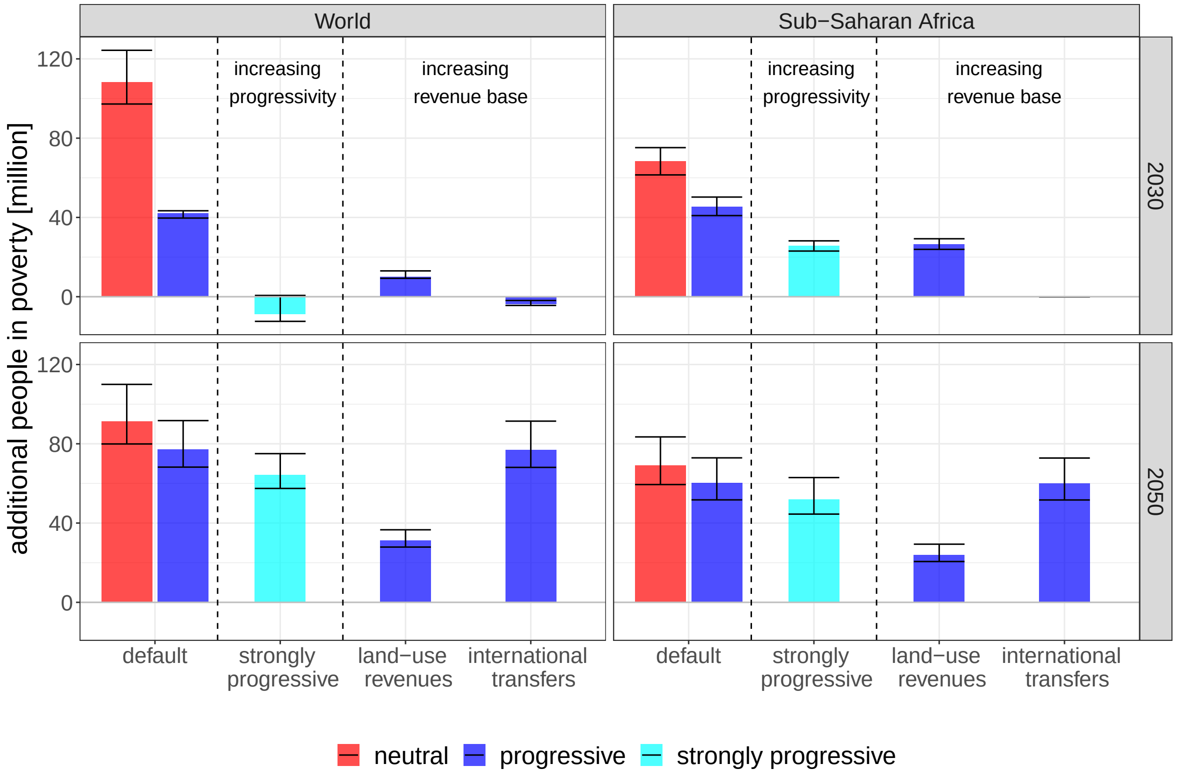

Supplementary Figure 12: *Additional* people in poverty for more progressive redistribution and different GHG pricing and burden sharing schemes: Same as Fig. 5 in the main text, but without the rescaling of food expenditures. The value for Sub-Saharan-Africa, 2030, international transfers is close to zero, such that the corresponding bar is not visible in the figure. See the caption of Fig. 4 in the main text for an explanation of the error bars.

the land-use related revenues, reduces the residual increase in poverty to around 25 million people in 2030. On the other hand, the international transfer scheme results in poverty trends approximately comparable to the baseline. Note however that unlike in our main analysis we do not find substantial net reductions in poverty through the policies explored here. Nonetheless, either a combination of the discussed three measures, or an increase in the transfer rate (beyond 5% of the carbon pricing revenues of industrialized countries) would still be an option to achieve this.

At the global level, already either of the two discussed additional measures would result in 2030 poverty outcomes comparable to the baseline. In 2050, on the other hand, we again find that additional funds for redistributive policies beyond the transfer of carbon pricing revenues would have to be sourced.

We conclude that our main finding, the ability to largely compensate the side effects of ambitious mitigation policies on poverty eradication at least in the near term, holds even under very pessimistic assumptions about the magnitude of food price effects. However, given the importance of food prices as a channel for distributional effects of climate policy in developing countries, further research on this topic is certainly warranted.

References

[1] Gunnar Luderer, Marian Leimbach, Nico Bauer, Elmar Kriegler, Lavinia Baumstark, Anastasis Giannousakis, Jérôme Hilaire, David Klein, Antoine Levesque, Michaja Pehl, Robert Pietzcker, Franziska Piontek, Niklas Roming, Valeria Jana Schwanitz, and Jessica Strefler. Description of the REMIND model (Version 1.6). 2015. URL https://www.pik-potsdam.de/research/transformation-pathways/models/remind/remind16_description_2015_11_30_final.

[2] Tino Aboumahboub, Cornelia Auer, Nico Bauer, Lavinia Baumstark, Christoph Bertram, Stephen Bi, Jan Dietrich, Alois Dirnaichner, Anastasis Giannousakis, Markus Haller, Jerome Hilaire, David Klein, Johannes Koch, Alexander Körner, Elmar Kriegler, Marian Leimbach, Antoine Levesque, Alexander Lorenz, Gunnar Luderer, Sylvie Ludig, Michael Lüken, Aman Malik, Susanne Manger, Leon Merfort, Ioanna Mouratiadou, Michaja Pehl, Robert Pietzker, Franziska Piontek, Laura Popin, Sebastian Rauner, Renato Rodrigues, Niklas Roming, Marianna Rottoli, Eva Schmidt, Felix Schreyer, Anselm Schultes, Björn Sörgel, Jessica Strefler, and Falko Ueckerdt. Remind - regional model of investments and development - version 2.1.0, 2020. URL https://www.pik-potsdam.de/research/transformation-pathways/models/remind.

[3] Jan Philipp Dietrich, Benjamin Leon Bodirsky, Florian Humpenöder, Isabelle Weindl, Miodrag Stevanović, Kristine Karstens, Ulrich Kreidenweis, Xiaoxi Wang, Abhijeet Mishra, David Klein, Geanderson Ambrósio, Ewerton Araujo, Amsalu Woldie Yalew, Lavinia Baumstark, Stephen Wirth, Anastasis Giannousakis, Felicitas Beier, David Meng-Chuen Chen, Hermann Lotze-Campen, and Alexander Popp. MAgPIE 4 – a modular open-source framework for modeling global land systems. *Geoscientific Model Development*, 12(4):1299–1317, April 2019. ISSN 1991-9603. doi: 10.5194/gmd-12-1299-2019. URL https://www.geosci-model-dev.net/12/1299/2019/.

[4] M. Meinshausen, S. C. B. Raper, and T. M. L. Wigley. Emulating coupled atmosphere-ocean and carbon cycle models with a simpler model, MAGICC6 – Part 1: Model description and calibration. *Atmospheric Chemistry and Physics*, 11(4):1417–1456, February 2011. ISSN 1680-7324. doi: 10.5194/acp-11-1417-2011. URL https://www.atmos-chem-phys.net/11/1417/2011/.

[5] Gunnar Luderer, Nico Bauer, Lavinia Baumstark, Christoph Bertram, Marian Leimbach, Robert Pietzcker, Jessica Strefler, Tino Aboumahboub, Cornelia Auer, Stephen Bi, Jan Dietrich, Alois Dirnaichner, Anastasis Giannousakis, Markus Haller, Jerome Hilaire, David Klein, Johannes Koch, Alexander Körner, Elmar Kriegler, Antoine Levesque, Alexander Lorenz, Sylvie Ludig, Michael Lüken, Aman Malik, Susanne Manger, Leon Merfort, Ioanna Mouratiadou, Michaja Pehl, Franziska Piontek, Laura Popin, Sebastian Rauner, Renato Rodrigues, Niklas Roming, Marianna Rottoli, Eva Schmidt, Felix Schreyer, Anselm Schultes, Bjoern Soergel, and Falko Ueckerdt. REMIND - REgional Model of INvestments and Development, May 2020. URL https://zenodo.org/record/3817324.

[6] Alexander Popp, Katherine Calvin, Shinichiro Fujimori, Petr Havlik, Florian Humpenöder, Elke Stehfest, Benjamin Leon Bodirsky, Jan Philipp Dietrich, Jonathan C. Doelmann, Mykola Gusti, Tomoko Hasegawa, Page Kyle, Michael Obersteiner, Andrzej Tabeau, Kiyoshi Takahashi, Hugo Valin, Stephanie Waldhoff, Isabelle Weindl,

Marshall Wise, Elmar Kriegler, Hermann Lotze-Campen, Oliver Fricko, Keywan Riahi, and Detlef P. van Vuuren. Land-use futures in the shared socio-economic pathways. *Global Environmental Change*, 42:331–345, January 2017. ISSN 09593780. doi: 10.1016/j.gloenvcha.2016.10.002. URL https://linkinghub.elsevier.com/retrieve/pii/S0959378016303399.

[7] Sibyll Schaphoff, Werner von Bloh, Anja Rammig, Kirsten Thonicke, Hester Biemans, Matthias Forkel, Dieter Gerten, Jens Heinke, Jonas Jägermeyr, Jürgen Knauer, Fanny Langerwisch, Wolfgang Lucht, Christoph Müller, Susanne Rolinski, and Katharina Waha. LPJmL4 – a dynamic global vegetation model with managed land – Part 1: Model description. *Geoscientific Model Development*, 11(4):1343–1375, April 2018. ISSN 1991-9603. doi: 10.5194/gmd-11-1343-2018. URL https://www.geosci-model-dev.net/11/1343/2018/.

[8] Benjamin Leon Bodirsky, Jan Philipp Dietrich, Eleonora Martinelli, Antonia Stenstad, Prajal Pradhan, Sabine Gabrysch, Abhijeet Mishra, Isabelle Weindl, Chantal Le Mouël, Susanne Rolinski, Lavinia Baumstark, Xiaoxi Wang, Jillian L. Waid, Hermann Lotze-Campen, and Alexander Popp. The ongoing nutrition transition thwarts long-term targets for food security, public health and environmental protection. *Scientific Reports*, 10(1):19778, November 2020. ISSN 2045-2322. doi: 10.1038/s41598-020-75213-3. URL https://www.nature.com/articles/s41598-020-75213-3. Number: 1 Publisher: Nature Publishing Group.

[9] Jan Philipp Dietrich, Florian Humpenöder, bodirsky, Kristine Karstens, mishkos, Abhijeet Mishra, David Klein, weindl, Xiaoxi Wang, Geanderson Ambrósio, Ewerton Araujo, FelicitasBeier, David Chen, and Anastasis Giannousakis. magpiemodel/magpie: MAgPIE 4.2.0, April 2020. URL https://zenodo.org/record/3752662.

[10] Joeri Rogelj, Daniel Huppmann, Volker Krey, Keywan Riahi, Leon Clarke, Matthew Gidden, Zebedee Nicholls, and Malte Meinshausen. A new scenario logic for the Paris Agreement long-term temperature goal. *Nature*, 573(7774):357–363, September 2019. ISSN 0028-0836, 1476-4687. doi: 10.1038/s41586-019-1541-4. URL http://www.nature.com/articles/s41586-019-1541-4.

[11] Joeri Rogelj, Drew Shindell, Kejun Jiang, Solomone Fifita, P Forster, Veronika Ginzburg, Collins Handa, Haroon Kheshgi, Shigeki Kobayashi, Elmar Kriegler, et al. Mitigation pathways compatible with 1.5°c in the context of sustainable development. In *Global Warming of 1.5°C. An IPCC Special Report on the impacts of global warming of 1.5°C above pre-industrial levels and related global greenhouse gas emission pathways, in the context of strengthening the global response to the threat of climate change, sustainable development, and efforts to eradicate poverty.* Intergovernmental Panel on Climate Change, 2018.

[12] Gunnar Myhre, Drew Shindell, et al. Anthropogenic and natural radiative forcing. In *Climate Change 2013: The Physical Science Basis. Contribution of Working Group I to the Fifth Assessment Report of the Intergovernmental Panel on Climate Change [Stocker, T.F., D. Qin, G.-K. Plattner, M. Tignor, S.K. Allen, J. Boschung, A. Nauels, Y. Xia, V. Bex and P.M. Midgley (eds.)].* Cambridge University Press, 2014.

[13] E. Calvo Buendia V. Masson-Delmotte H.-O. Pörtner D. C. Roberts P. Zhai R. Slade S. Connors R. van Diemen M. Ferrat E. Haughey S. Luz S. Neogi M. Pathak J. Petzold J. Portugal Pereira P. Vyas E. Huntley K. Kissick M. Belkacemi J. Malley (eds.) P.R. Shukla,

J. Skea. Summary for policymakers. In *Climate Change and Land: an IPCC special report on climate change, desertification, land degradation, sustainable land management, food security, and greenhouse gas fluxes in terrestrial ecosystems*. Intergovernmental Panel on Climate Change, 2019.

[14] Paul L. Lucas, Detlef P. van Vuuren, Jos G.J. Olivier, and Michel G.J. den Elzen. Long-term reduction potential of non-CO2 greenhouse gases. *Environmental Science & Policy*, 10(2):85–103, April 2007. ISSN 1462-9011. doi: 10.1016/j.envsci.2006.10.007. URL http://www.sciencedirect.com/science/article/pii/S1462901106001316.

[15] Eric Kemp-Benedict. Income distribution and poverty: methods for using available data in global analysis. Polestar Technical Note No.4:16, 2001.

[16] Humberto Lopez and Luis Serven. *A Normal Relationship ? Poverty, Growth, And Inequality*. Policy Research Working Papers. The World Bank, January 2006. doi: 10.1596/1813-9450-3814. URL http://elibrary.worldbank.org/doi/book/10.1596/1813-9450-3814.

[17] John Aitchison and James AC Brown. The lognormal distribution with special reference to its uses in economics. 1957.

[18] Thomas Sterner. *Fuel taxes and the poor: the distributional effects of gasoline taxation and their implications for climate policy*. Routledge, 2012.

[19] World Bank. Global consumption database, 2020. http://datatopics.worldbank.org/consumption/AboutDatabase.

[20] Ira Irina Dorband, Michael Jakob, Matthias Kalkuhl, and Jan Christoph Steckel. Poverty and distributional effects of carbon pricing in low- and middle-income countries – A global comparative analysis. *World Development*, 115:246–257, March 2019. ISSN 0305750X. doi: 10.1016/j.worlddev.2018.11.015. URL https://linkinghub.elsevier.com/retrieve/pii/S0305750X18304212.

[21] World Bank. World development indicators, 2020. https://datacatalog.worldbank.org/dataset/world-development-indicators.

[22] Jewell, J. and McCollum, D. Report on improving the representation of existing energy policies (taxes and subsidies) in iams (advance deliverable no. 3.1), 2014. http://www.fp7-advance.eu/?q=content/subsidy-and-energy-tax-dataset.

[23] Andrew Muhammad, James L. Seale, Birgit Meade, and Anita Regmi. International Evidence on Food Consumption Patterns: An Update Using 2005 International Comparison Program Data. *SSRN Electronic Journal*, 2011. ISSN 1556-5068. doi: 10.2139/ssrn.2114337. URL http://www.ssrn.com/abstract=2114337.

[24] Nico Bauer, Christoph Bertram, Anselm Schultes, David Klein, Gunnar Luderer, Elmar Kriegler, Alexander Popp, and Ottmar Edenhofer. Quantification of an efficiency–sovereignty trade-off in climate policy. *Nature*, 588(7837):261–266, December 2020. ISSN 1476-4687. doi: 10.1038/s41586-020-2982-5. URL https://www.nature.com/articles/s41586-020-2982-5. Number: 7837 Publisher: Nature Publishing Group.

[25] Matthias Kalkuhl and Leonie Wenz. The impact of climate conditions on economic production. evidence from a global panel of regions. *Journal of Environmental Economics and Management*, 103:102360, 2020. ISSN 0095-0696. doi: https://doi.org/10.1016/j.jeem.2020.102360. URL http://www.sciencedirect.com/science/article/

pii/S0095069620300838.

[26] Narasimha D. Rao, Petra Sauer, Matthew Gidden, and Keywan Riahi. Income inequality projections for the Shared Socioeconomic Pathways (SSPs). *Futures*, August 2018. ISSN 00163287. doi: 10.1016/j.futures.2018.07.001. URL https://linkinghub.elsevier.com/retrieve/pii/S001632871730349X.

[27] Narasimha D Rao and Shonali Pachauri. Energy access and living standards: some observations on recent trends. *Environmental Research Letters*, 12(2):025011, February 2017. ISSN 1748-9326. doi: 10.1088/1748-9326/aa5b0d. URL http://stacks.iop.org/1748-9326/12/i=2/a=025011?key=crossref.695ec63a546756c629d9924c69c1a863.

[28] Aziz Atamanov, R. Andres Castaneda Aguilar, Tony H.M.J. Fujs, Reno Dewina, Carolina Diaz-Bonilla, Daniel Gerszon Mahler, Dean Jolliffe, Christoph Lakner, Mikhail Matytsin, Jose Montes, Laura L. Moreno Herrera, Rose Mungai, David Newhouse, Minh C. Nguyen, Francisco J. Parada Gomez Urquiza, Ani Rudra Silwal, Diana M. Sanchez Castro, Marta Schoch, David L. Vargas Mogollon, Martha C. Viveros Mendoza, Judy Yang, Nobuo Yoshida, and Haoyu Wu. *March 2020 PovcalNet Update: What's New*. World Bank, March 2020. doi: 10.1596/33496. URL http://elibrary.worldbank.org/doi/book/10.1596/33496.

[29] Spencer L James, Paul Gubbins, Christopher JL Murray, and Emmanuela Gakidou. Developing a comprehensive time series of GDP per capita for 210 countries from 1950 to 2015. *Population Health Metrics*, 10(1), December 2012. ISSN 1478-7954. doi: 10.1186/1478-7954-10-12. URL http://pophealthmetrics.biomedcentral.com/articles/10.1186/1478-7954-10-12.

[30] World Bank. Poverty and shared prosperity 2018: Piecing together the poverty puzzle, 2018.
