# Supplementary material for: Combining ambitious climate policies with efforts to eradicate poverty
Source: Nat Commun. 2021 Apr 27;12:2342. doi: 10.1038/s41467-021-22315-9 (PMC8079366; doi:10.1038/s41467-021-22315-9)
Supplement: Supplementary file 3 — Reporting Summary [file 41467_2021_22315_MOESM3_ESM.pdf]

nature research

Corresponding author(s): Bjoern Soergel
Last updated by author(s): Feb 9, 2021

Reporting Summary

Nature Research wishes to improve the reproducibility of the work that we publish. This form provides structure for consistency and transparency in reporting. For further information on Nature Research policies, see our Editorial Policies and the Editorial Policy Checklist.

Statistics

For all statistical analyses, confirm that the following items are present in the figure legend, table legend, main text, or Methods section.

n/a	Confirmed	
☐	☒	The exact sample size (*n*) for each experimental group/condition, given as a discrete number and unit of measurement
☒	☐	A statement on whether measurements were taken from distinct samples or whether the same sample was measured repeatedly
☐	☒	The statistical test(s) used AND whether they are one- or two-sided<br>*Only common tests should be described solely by name; describe more complex techniques in the Methods section.*
☐	☒	A description of all covariates tested
☒	☐	A description of any assumptions or corrections, such as tests of normality and adjustment for multiple comparisons
☐	☒	A full description of the statistical parameters including central tendency (e.g. means) or other basic estimates (e.g. regression coefficient) AND variation (e.g. standard deviation) or associated estimates of uncertainty (e.g. confidence intervals)
☐	☒	For null hypothesis testing, the test statistic (e.g. *F*, *t*, *r*) with confidence intervals, effect sizes, degrees of freedom and *P* value noted<br>*Give P values as exact values whenever suitable.*
☒	☐	For Bayesian analysis, information on the choice of priors and Markov chain Monte Carlo settings
☒	☐	For hierarchical and complex designs, identification of the appropriate level for tests and full reporting of outcomes
☒	☐	Estimates of effect sizes (e.g. Cohen's *d*, Pearson's *r*), indicating how they were calculated

Our web collection on statistics for biologists contains articles on many of the points above.

Software and code

Policy information about availability of computer code

Data collection	The IAM scenario data analysed in this paper is computed with the open-source IAM framework REMIND-MAgPIE (model versions: REMIND 2.1.1, MAgPIE 4.2)
Data analysis	Data analysis was performed in R (version 3.6.3), relying on widely used open-source libraries (e.g. "tidyverse"). R-libraries that are specifically built for processing REMIND-MAgPIE output are also available open source (github.com/pik-piam).

For manuscripts utilizing custom algorithms or software that are central to the research but not yet described in published literature, software must be made available to editors and reviewers. We strongly encourage code deposition in a community repository (e.g. GitHub). See the Nature Research guidelines for submitting code & software for further information.

Data

Policy information about availability of data

All manuscripts must include a data availability statement. This statement should provide the following information, where applicable:

- Accession codes, unique identifiers, or web links for publicly available datasets
- A list of figures that have associated raw data
- A description of any restrictions on data availability

The IAM scenario data analysed in this paper are computed with models that are available open source (see SI for links to code repositories).
The data on energy and food expenditures by income group (see Section 5.2 of SI for details) were kindly provided by the authors of Dorband et al., 2019 [Ref. 33], who in turn derived them from the World Bank's Global Consumption Database [Ref. 70].
The data shown in the main figures of this study are available in this repository: https://doi.org/10.5281/zenodo.4320973 .
Intermediate data sets generated or analysed in this study are available from the corresponding author on reasonable request.

Field-specific reporting

Please select the one below that is the best fit for your research. If you are not sure, read the appropriate sections before making your selection.

☐ Life sciences ☒ Behavioural & social sciences ☐ Ecological, evolutionary & environmental sciences

For a reference copy of the document with all sections, see nature.com/documents/nr-reporting-summary-flat.pdf

Behavioural & social sciences study design

All studies must disclose on these points even when the disclosure is negative.

Study description	Quantitative analysis of the consequences of ambitious climate policies for the goal to eradicate poverty. The study is based on IAM scenario data, but involves two elements relying on existing empirical data : (1) a regression model linking average income and levels of inequality to poverty rates, (2) an analysis of the distributional effects of rising energy and food prices. The responses given below refer to these two empirical analyses.
Research sample	(1) country-year observations of Gini coefficients and poverty rates for nearly all countries, years 1990-2015. Obtained from World Development Indicators (https://datatopics.worldbank.org/world-development-indicators/), indicator codes SI.POV.GINI and SI.POV.DDAY.<br>(2) aggregated energy and food expenditures for four different income groups in around 80 countries, year around 2010. Processed as described in Dorband et al. 2019 [Ref. 33], original data from Global Consumption Database (https://datatopics.worldbank.org/consumption/). Kindly provided by the authors of Dorband et al.
Sampling strategy	All available data was used.
Data collection	Does not apply since this study did not involve the collection of any additional data; there were no participants involved. Only existing data (e.g. from the World Bank's World Development Indicators, see above) was used.
Timing	Does not apply since this study did not involve the collection of any additional data
Data exclusions	Countries with no available data on poverty rates where excluded from the analysis (1).
Non-participation	Does not apply since this study did not involve the collection of any additional data; there were no participants involved.
Randomization	Does not apply since this study did not involve the collection of any additional data; there were no participants involved.

Reporting for specific materials, systems and methods

We require information from authors about some types of materials, experimental systems and methods used in many studies. Here, indicate whether each material, system or method listed is relevant to your study. If you are not sure if a list item applies to your research, read the appropriate section before selecting a response.

Materials & experimental systems

n/a	Involved in the study
☒	☐ Antibodies
☒	☐ Eukaryotic cell lines
☒	☐ Palaeontology and archaeology
☒	☐ Animals and other organisms
☒	☐ Human research participants
☒	☐ Clinical data
☒	☐ Dual use research of concern

Methods

n/a	Involved in the study
☒	☐ ChIP-seq
☒	☐ Flow cytometry
☒	☐ MRI-based neuroimaging
